# Oral Health Status in Three Long Term Care Units of Schizophrenic Patients in Chaharmahal-Bakhtiari Province, Iran

**DOI:** 10.5812/ircmj.1892

**Published:** 2013-04-05

**Authors:** Masood Nikfarjam, Neda Parvin

**Affiliations:** 1 Department of Medicine, Shahrekord University of Medical Sciences, Shahrekord, IR Iran; 2 Department of Nursing, Shahrekord University of Medical Sciences, Shahrekord, IR Iran

**Keywords:** Schizophrenia, Oral Health

*Dear Editor,*

People with serious mental illness experience an erosion of functioning in day-to-day life over a protracted period of time. There is also evidence to suggest that people with serious mental illness have a greater risk of experiencing oral disease and have greater oral treatment needs than the general population. However, oral health has never been seen as a priority in people suffering with serious mental illness. Poor oral health has a serious impact on quality of life, everyday functioning, social inclusion and self-esteem (1). There are some evidences that the patients with schizophrenia show poor oral health and great need for periodontal treatment. The goal of this cross-sectional study was to assess the oral health status of a sample institutionalized patients with schizophrenia in Iran.

The subjects were selected from patients with schizophrenia in three psychiatric long term care units in Chaharmala- Bakhtiary province, Iran during 2008. The inclusion criteria were patients with documented diagnosis of schizophrenia (DSM-IV-TR). The data were analyzed by Mann-Whitney and Spearman correlation test. The study was approved by Ethical Committee of Shahrekord University of Medical Sciences. The data were gathered by questionnaire for demographic information, patients' medical sheets, psychological interview and completing Standard Anderson Positive and Negative Scale questionnaires (SAPS, SANS), and complete dental examination determining Decayed/Missing/Filled Teeth (DMFT) index of the total 123 schizophrenic patients. In this study, the average age of patients were 38.81 ± 10.46 years old, ninety percent had an education level of under diploma, most of them were urban residents, 82.8% were unemployed, and 67% had a history of hospitalization, mean hospitalization period was 6.67 ± 5.37 years, mean number of cigarettes per day was 3.95 ± 5.48, and mean smoking period was 7.20 ± 9.08 years.

Mean score of positive and negative symptoms in patients was 97.27 ± 18.90 and 77.65 ± 14.45 respectively and mean DMFT was 19.43 ± 7.71, with a mean number of 11.24 ± 6.97 decayed, 8.17 ± 8.30 missing and 1.1 ± 0.41 filled teeth. The Spearman test showed a significant positive correlation between age, smoking period, mean number of cigarettes per day and mean hospitalization period. The DMFT significantly increased with age (P = 0.001, r = 0.527), smoking period (P = 0.001, r = 0.332), mean number of cigarettes per day (P = 0.031, r = 0.171) and mean hospitalization period (P = 0.043, r = 0.157), but Mann-Whitney test showed no significant relation between sex and positive symptoms with DMFT (P > 0.05). In addition, the Spearman test showed SANS (P = 0.035, r = 0.142) has a significant positive correlation with DMFT and the DMFT in persons who had a severe negative symptoms was worse than others. We found a mean number of 11.24 ± 6.97 decayed teeth, 8.17 ± 8.30 missing teeth and almost no filling teeth in our study population which is in consistent with the findings of Velasco study reporting a mean number of 7.9, 17 and 0 for decayed, missing and filled teeth respectively (2). Both findings reveal poor dental health status in schizophrenic patients, they not receiving treatments such as filling but instead extracting the decayed teeth.

McCreadie and Arnaiz also mentioned that there is a positive correlation between negative symptoms of the schizophrenia and DMFT (3, 4). These findings support Gurbuz and colleagues findings and also demonstrate the need for further efforts to prevent oral diseases among patients with severe mental illness (5). Our results also confirmed this positive correlation which is mostly due to the nature of the negative symptoms leading to lack of motivation and social isolation. Negative symptoms such as apathy and loss of drive may lead to poor self-care. In this context it is noteworthy that those who did not brush their teeth regularly had more negative symptoms as assessed by the SANS. In other hand, most of patients were smokers and smoking can contribute to poor dental health (6). According to the findings of this study, the oral and dental health status is very poor among chronic schizophrenic patients. These underline the urgent need for specific preventive oral health programs to improve the dental care of these patients and mental health professionals should pay more attention to preventive oral health habits of psychiatric in patients.
